# Spinal Cord Pathology in Alpha-Synuclein Transgenic Mice

**DOI:** 10.4061/2010/375462

**Published:** 2010-07-08

**Authors:** Sonja Mendritzki, Saskia Schmidt, Teresa Sczepan, Xin-Ran Zhu, Daniel Segelcke, Hermann Lübbert

**Affiliations:** ^1^Department of Animal Physiology, Biology, and Biotechnology, Ruhr-University Bochum, ND/5131, D-44780 Bochum, Germany; ^2^International Graduate School of Neuroscience (IGSN), Ruhr-University Bochum, 44780 Bochum, Germany

## Abstract

Accumulation of *α*-synuclein is observed in neurodegenerative diseases like Parkinson's disease and Multiple System Atrophy. In previous studies with transgenic C57BL/6 mice overexpressing *α*-synuclein carrying the mutations A53T and A30P found in Parkinson's disease or with a parkin-null background, we reported severe mitochondrial impairments in neurons and to a larger extent in glial cells of the mesencephalon. Neuron death was not observed in the brain. Here we show that the mice show severe motor impairments in behavioral tests. In addition, these mice exhibit astrocytic cell death in the spinal cord, accompanied by extensive gliosis and microglial activation. This is shown by cell death staining and immunohistochemistry. Ultrastructural analyses revealed severe mitochondrial impairments not only in astrocytes, but also in oligodendrocytes and, to a small extent, in neurons. Thus, the transgenic mice show a profound pathology in glial cells of the spinal cord.

## 1. Introduction

Parkinson's disease (PD) is a chronic neurodegenerative disease associated with severe motor impairments, substantial morbidity, and increased mortality. The neuropathology of PD is characterized by an excessive loss of dopaminergic neurons in the substantia nigra, but other brain regions may also be affected. Most cases of PD are sporadic, with late onset. However, 5–10% of PD cases are familial and can be attributed to a mutation in genes like *α*-synuclein (*α*-syn) and parkin [1]. *α*-syn is a synaptic molecule and it is assumed that it is involved in membrane vesicle trafficking and cytoskeletal dynamics [2]. Furthermore, aggregations of *α*-syn are specific for *α*-synuncleinopathies like PD, Lewy body disease, or Multiple System Atrophy (MSA) [3, 4]. It seems to be that *α*-syn is degraded by Parkin, an E3 ubiquitin ligase [5].

Several transgenic mouse models carrying *α*-syn and parkin mutations have been used to elucidate the molecular mechanisms of PD pathogenesis [6]. In previous studies, we generated mice carrying a deletion in the parkin gene and/or overexpressing the mutated human *α*-syn transgene [7, 8]. These mice exhibit ultrastructural mitochondrial alterations in neurons and, interestingly, to a larger extent in glial cells of the mesencephalon, but they do not display severe histopathological hallmarks such as cell death in the substantia nigra. These data indicate that glial cells are active contributors to the initiation or progression of PD [9, 10]. This is supported by cell culture data suggesting abnormal functions of glial cells from mice carrying PD-inducing gene mutations [11, 12]. Furthermore, glial cells have been shown to express proteins like superoxide dismutase 1, huntingtin, or ataxin which are linked to neurodegenerative diseases. Recent studies suggest an important role for glial cells also in other neurodegenerative diseases. For example, astrocytes play a central role in the degeneration of spinal motor neurons in amyotrophic lateral sclerosis [13] and oligodendrocytes are active contributors to neuronal degeneration in MSA [14, 15]. 

Several clinical studies reported the appearance of nondopaminergic symptoms like impairments in anxiety behavior in early PD stages [16]. Recent literature suggests that pathologic manifestations are first detected in the spinal cord and then progressing from caudal to rostral until they reach the mesencephalon. This model, described by Braak and coworkers [17], provides an explanation for the appearance of nondopaminergic symptoms which precede cell death in the substantia nigra. Autonomic dysfunctions like depression, sleep disturbances, or anxiety behavior are controlled by the lower brainstem and spinal ganglia [18]. 

Based on these reports, we now investigated whether a caudal to rostral progression could also be observed in our transgenic mice, since parkin and *α*-syn are expressed in the spinal cord [19]. We found cell death, mitochondrial alterations, and severe inflammatory reactions in glial cells of the spinal cords of the mutated mice. In addition, when looking at the motor abilities of the mice, we found severe motor impairments which seem to be related to the pathological changes in the spinal cord.

In summary, our transgenic mice carrying PD-inducing gene mutations exhibit more severe pathological impairments in glial cells of the spinal cord than in the mesencephalon, similar to the situation in early PD described by Braak and coworkers [17].

## 2. Methods

### 2.1. Mouse Strains

Two transgenic mouse lines were analyzed: a homozygous *α*-syn line (C57BL/6J background) overexpressing doubly mutated (A30P, A53T) human (hm^2^) *α*-syn driven by the chicken *β*-actin (BAsyn) promoter as described previously [7], here we used the BAsyn line 1.5 and a double-mutant mouse line carrying additionally a deletion of exon3 in the parkin gene (BAsyn/PaKO; F10 generation, 99% C57BL/6J, and 1% 129SvJ). Control mice were age-matched nontransgenic littermates (LMs). 

Housing and breeding of animals were performed in accordance with the German guidelines of the animal care and use committee. All efforts were made to minimize the number of animals used and their suffering.

### 2.2. Tissue Preparation

Mice were anesthetized by an overdose of ketamine (100 mg/kg) and rompun (5 mg/kg) and perfused transcardially with phosphate-buffered saline (PBS) followed by 4% paraformaldehyde (PFA) in 0.1 M phosphate buffer (PB) for immunohistochemistry or 2% PFA plus 2.5% glutaraldehyde (GA) in PB for electron microscopy. Cervical parts of the spinal cord were immediately removed and postfixed.

### 2.3. Immunohistochemistry and Cell Death Staining

Cervical parts of the spinal cord were embedded in paraffin. For sequential double-labeling spinal cords were cut into 3 *μ*m-thick sections, for analyses of *α*-syn aggregations, for cell death staining as well as for the investigations of inflammatory processes spinal cords were cut into 18 *μ*m-thick sections. The sections were deparaffinized and antigen retrieval (5–10 min cooking in 0.01 M citrate buffer, pH 6.0) was performed (except for glial fibrillary acidic protein (GFAP) and myelin basic protein (MBP)). After preincubation (3% normal serum), sections were treated with mouse antihuman *α*-syn (anti-syn211; 1 : 700, Invitrogen), astrocyte marker rabbit anticow GFAP (anti-GFAP; 1 : 1000; Acris), oligodendrocyte marker goat antihuman MBP (anti-MBP; 1 : 1000; Santa Cruz), microglia marker goat antihuman ionized calcium-binding adaptor molecule 1 (anti-Iba1; 1 : 250; Abcam), neuronal marker mouse antimouse neuronal nuclei (anti-NeuN; 1 : 700; Chemicon), goat antimouse cathepsin X (anti-CATX; 1 : 200; R&D Systems), and goat antirat cathepsin S (anti-CATS; 1 : 200; Santa Cruz). Sections were stained with the corresponding biotinylated secondary antibody (1 : 300, Axxora), ABC reagent (1 : 100; Axxora), and silver-gold intensification. 

FluoroJade and silver-staining were used to detect degenerating cells. Deparaffinized and rehydrated sections were incubated in 0.06% potassium permanganate solution for 15 min, rinsed with distilled water, and then incubated in a solution of 0.01% FluoroJade (Histo-Chem) in 0.1% acetic acid for 30 min. Silver staining was performed according to the protocol of Gallyas et al. [20].

### 2.4. Transmission Electron Microscopy

Postfixed (2 h, 4°C) parts of the cervical spinal cord were cut with a vibratome (100 *μ*m thick), anterior horn samples microdissected and placed in 2% OsO4 for 1 h, dehydrated with graded concentrations of ethanol and Epon-propylene oxide, and flat embedded in Epon. Representative ultrathin sections were collected on Formvar-coated grids and contrasted with uranyl acetate and lead citrate.

Ultrathin sections were visualized using a Phillips EM-410. Morphology and number of mitochondria were evaluated in digitized electron microscopic images of 8 m-old animals. In total, 24 somata per cell type (neurons, astrocytes, and oligodendrocytes) taken from two vibratome sections of each animal were independently analyzed by two examiners blind with respect to the genotypes of the mice. Ultrathin sections were selected at random from the middle of the vibratome sections to avoid potential structural irregularities possibly occurring on the surface. 

Data are presented as a mean ± standard deviation (SD) of the means of the analyzed animals per genotype. Statistical analyses of ultrastructural changes compared to corresponding age-matched LMs were carried out using the Mann-Whitney Rank Sum Test (*U*-test, two tailed, alpha level 0.05).

### 2.5. Elevated Plus Test

Mice were placed on a cross-shaped elevated maze with two open arms and two closed arms which stands one meter above the floor. A video camera recorded for 5 min the horizontal path length, time in closed arms, and duration and number of stops.

### 2.6. Footprints

To obtain the footprints, the hind paws of the mice were dipped in a dye and the mice were placed in a 100 cm long and 4.5 cm wide gangway. The floor was lined with white paper and the footprints were analyzed with a Footprint software. Data are presented as mean ± SD of the means of the analyzed animals per genotype. Statistical analyses were performed by using the Mann-Whitney Rank Sum Test (*U*-test, two tailed, alpha level 0.05).

## 3. Results

### 3.1. Cellular Distribution of the hm *α*-Syn in the Spinal Cord of BAsyn and BAsyn/PaKO

**Figure 1:**
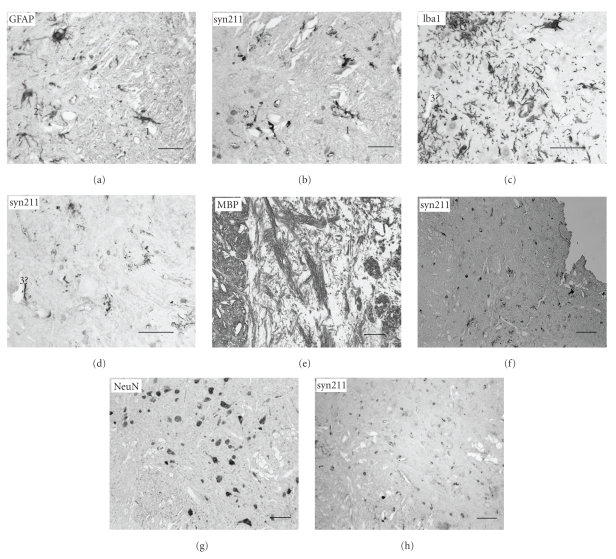
Light microscopic phenotyping of cells expressing the transgene in BAsyn/PaKO mouse brains. Photomicrographs of serial, 3 *μ*m-thick sections of the spinal cord from BAsyn/PaKO mice ((a)–(h)). The syn211-immunostaining (b) colocalized with the marker GFAP (a), indicating the transgene expression in astrocytes. In the parts of the spinal cord where many Iba1-positive cells (c) are stained, it seems that only one cell could be also positive for syn211 (d), indicating that it is not clear if the hm *α*-syn is expressed in microglia. For MBP-immunopositive oligodendrocytes (e) and NeuN-immunopositive (g) neurons no match with syn211-immunostaining could be detected ((f), (h)). Scale bars: (a)–(d): 30 *μ*m; (e)–(h) 50 *μ*m.

**Figure 2:**
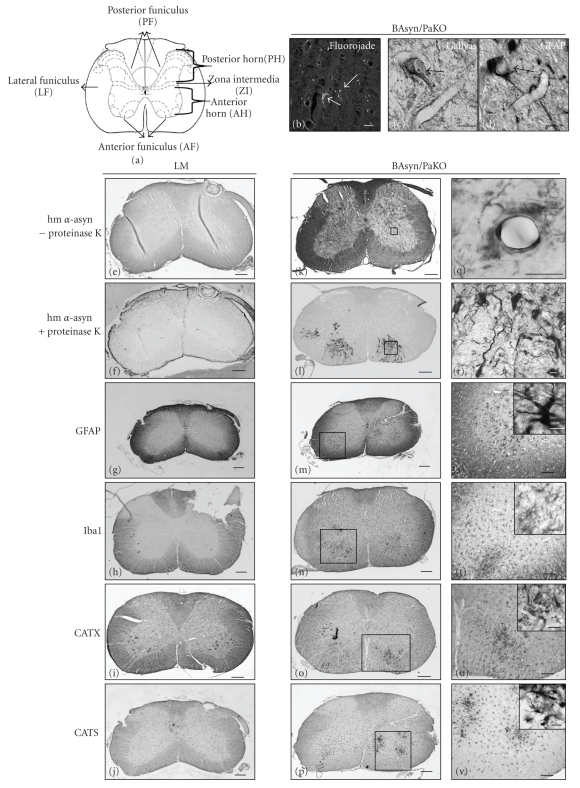
Immunohistochemical analyzes and cell death staining of the cervical spinal cord. Schematic illustration of the transversal spinal cord (a). Cell death staining by FluoroJade and Gallyas are shown in (b) and (c) for BAsyn/PaKO. An example showed a cell positive for Gallyas and GFAP in adjacent slices ((c), (d)). Immunopositive structures of the cervical spinal cord for hm *α*-syn (with or without proteinase K), GFAP, Iba1, CATX, and CATS are shown for LM in (e)–(j) and for BAsyn/PaKO in (k)–(p). (q)–(v) are higher magnifications of (k)–(p). Scale bars: (e)–(p): 200 *μ*m; (q) and (r): 10 *μ*m, (s)–(v): 100 *μ*m, and details of (s)–(v): 10 *μ*m.

 We investigated and compared the distribution of the human *α*-syn in the transgenic *α*-syn mouse lines. Sequential immunoperoxidase labeling with *α*-syn- and cell type-specific antibodies was performed. Examples are shown for 8-month (m) old BAsyn/PaKO in Figures 1(a)–1(h). BAsyn/PaKO expressed the transgene in the spinal cord almost exclusively in astrocytes (Figures 1(a) and 1(b)). In Figure 2(k), the *α*-syn distribution is shown for a whole cross section of the spinal cord from BAsyn/PaKO, indicating that the transgene is mostly expressed in white matter. In this area we also found many GFAP-positive cells (Figure 2(l)). It is not clear if the transgene is expressed in microglia. The parts of the spinal cord with a large amount of microglia showed nearly no *α*-syn expression (Figures 1(c) and 1(d)). However, it cannot be excluded that the transgene is expressed in few isolated microglia. Neither for immunopositive oligodendrocytes nor for stained neurons an expression of the transgene could be detected (Figures 1(e)–1(h)). We found the same cellular distribution in BAsyn mice (data not shown).

### 3.2. Severe *α*-Syn Aggregations, Inflammatory Reactions, and Cell Death in the Spinal Cord of *α*-Syn Transgenics

 We analyzed the cervical spinal cord of 3- and 8-m old BAsyn and BAsyn/PaKO mice and their corresponding LM as illustrated in Figure 2. We examined the expression of the hm *α*-syn without (Figures 2(s), 2(k), and 2(q)) and with (Figures 2(f), 2(l), and 2(r)) pretreatment of the slices with proteinase K. After pretreatment of the slices with proteinase K hm, *α*-syn aggregations in the anterior horn (AH), zona intermedia (ZI), lateral funiculus (LF), and in the anterior funiculus (AF) of the spinal cord were visible in 8 m-old BAsyn/PaKO (Figures 2(l) and 2(r)). In contrast, no aggregations were observed in LM (Figure 2(f)). 

Next, we investigated the expression of GFAP and Iba1 and found an increased number of GFAP-positive cells (Figures 2(m) and 2(s)) as well as a stronger expression of Iba1 (Figures 2(n) and 2(t)) in the AH, ZI, LF, and in the AF of the spinal cord of 8 m-old BAsyn/PaKO compared to LM (Figures 2(g) and 2(h)), indicating the occurrence of severe gliosis. 

This glial cell activation in the old BAsyn/PaKO was accompanied by a higher expression of the lysosomal cystein proteases CATX (Figures 2(o) and 2(u)) and CATS (Figures 2(p) and 2(v)). Expression of these markers is an early indicator of inflammatory reactions. The two proteins were visible in the same regions where gliosis was observed, different from the LM (Figures 2(i) and 2(j)).

In addition to the severe *α*-syn aggregations, gliosis and inflammation, FluoroJade (Figure 2(b)) and Gallyas silver staining (Figure 2(c)) indicated the occurrence of cell death in the LF and AF of the cervical spinal cord of 8 m-old BAsyn/PaKO. Analyses of adjacent slices showed that the cells positive for Gallyas cell death staining were also stained by astrocytes markers (Figures 2(c), 2(d)). No cell death could be detected in motor neurons located next to the astrocytes.

The same analyzes were performed with the monomutant line BAsyn and in two out of four animals we also found *α*-syn aggregations, gliosis, inflammatory reactions as well as cell death. However, two other animals did not display these pathological changes (data not shown). Furthermore, 3 m-old mice were investigated and neither young BAsyn nor BAsyn/PaKO displayed these alterations (data not shown).

### 3.3. Ultrastructural Impairments of Mitochondria in the Spinal Cord of the Double Mutants

**Figure 3:**
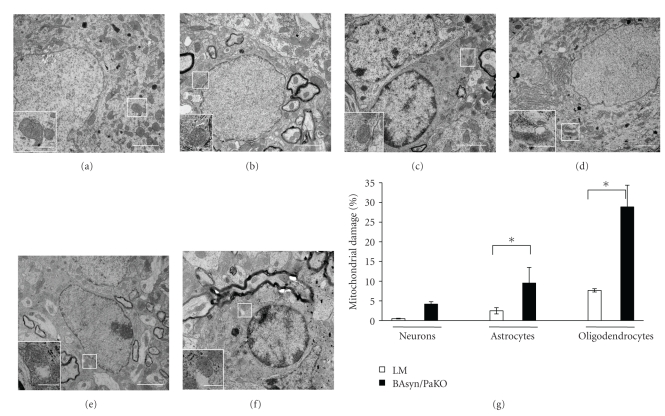
Mitochondrial ultrastructure in the spinal cord of 8 m-old BAsyn/PaKO. Exemplary ultrastructural features of neurons, astrocytes, and oligodendrocytes are shown for LM ((a)–(c)) and BAsyn/PaKO ((d)–(f)). Higher magnifications show examples of healthy or damaged mitochondria. Scale bars: (a)–(f): 2 *μ*m; higher magnification of (a)–(f): 0.5 *μ*m. The quantification of mitochondrial alterations is shown in (g), *n* = 3, errors bars = SD, *U*-test, *P < .05.

 The mitochondrial ultrastructure of neurons, astrocytes, and oligodendrocytes in the cervical spinal cord was examined by electron microscopy. Glial cells were counted if they contained a nucleus surrounded by cytoplasm and were classified according to their typical ultrastructural features [21, 22]. Briefly, astrocytes were identified by their electron-lucent cytoplasm and the appearance of their nuclei (thin rim of heterochromatin adjacent to nuclear membrane). Oligodendrocytes show electron-dense cytoplasms and their nuclei are rich in clumped heterochromatin close to the border of the nucleus. Mitochondria were classified as structurally damaged when one or more of the following alterations were observed: distorted or disrupted cristae, detachment of or protrusions from the outer membrane, electron-lucent domains, or inclusions in the matrix. At the age of 8 m BAsyn/PaKO showed only a slight mitochondrial damage in neurons (Figures 3(d) and 3(g)), whereas LM exhibited nearly no impairments (Figures 3(a) and 3(g)). In contrast, both astrocytes (Figures 3(e) and 3(g)) and oligodendrocytes (Figures 3(e) and 3(g)) of BAsyn/PaKO displayed a statistically significant increase in mitochondrial damage compared to the corresponding cells in LM (Figures 3(b), 3(c), and 3(g)). Oligodendrocytes exhibited the highest number of altered mitochondria.

### 3.4. Behavioral Deficits in Mono- and Double-Mutant Mice

**Figure 4:**
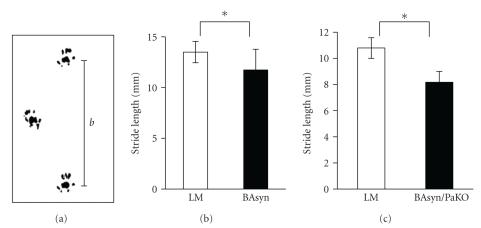
Footprint analyzes of BAsyn and BAsyn/PaKO. Footprints (a) and stride length ((b), (c)). The stride lengths of 8–10 mice per group were determined, error bars = SD, *U*-test, *P < .05.

 The gait of the 8 m-old transgenic mice was investigated by analyzing their footprints. The following parameters were measured: stride length, stride width, foot length, area touched, angle, and spread of toes 1–5 and 2–4 (Figure 4(a)). Examinations revealed a decreased stride length for both mono- and double-mutants (Figures 4(b)–4(c)). 

**Figure 5:**
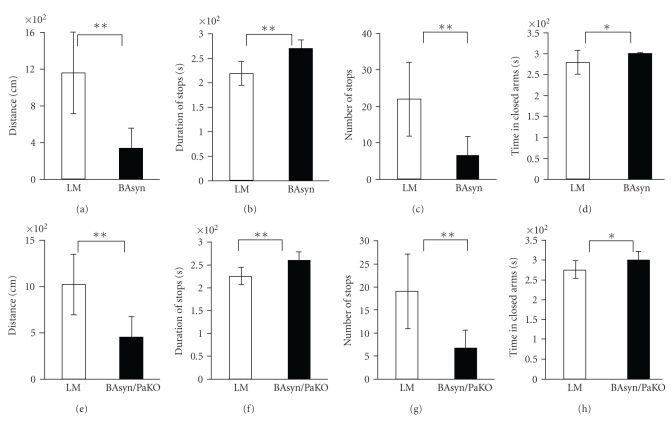
Elevated plus test performance of 8 m-old BAsyn and BAsyn/PaKO. Walked distance ((a), (e)), duration of stops ((b), (f)), number of stops ((c), (g)), and time spent in closed arms ((d), (h)) are shown, *n* = 6–9, error bars = SD, *U*-test, *P < .05, **P < .01.

The Elevated Plus Test measures the anxiety as well as the willingness of mice to explore a new environment. Four different parameters were analyzed: distance, time in closed arms, number of stops, and the duration of stops. Both 8 m-old BAsyn and BAsyn/PaKO walked shorter distances made fewer stops and longer breaks than LM. The transgenic mice spent by trend more time in the closed arms, however, this difference is very low (Figures 5(a)–5(h)). 

## 4. Discussion

While the pathological hallmark of PD is the degeneration of dopaminergic neurons in the substantia nigra, it has recently been suggested that dopaminergic neuron death is preceded by pathological changes in more caudal CNS regions [17]. It has been proposed that such alterations are accompanied by *α*-syn aggregations in the spinal cord [17, 23]. These findings raise new questions about the starting point of PD pathology. 

We are analyzing transgenic mouse models overexpressing hm^2^ *α*-syn alone (BAsyn) or in combination with a deletion of the exon3 of the parkin gene (BAsyn/PaKO). As these mice do not exhibit gross pathological impairments in the mesencephalon we investigated in this study the cervical part of the spinal cord according to the staging scheme of Braak and coworkers [17, 23].

We first demonstrated for both mouse lines the expression of hm^2^ *α*-syn in the spinal cord almost exclusively in astrocytes. For neurons, microglia, and oligodendrocytes no *α*-syn expression could be detected. 

Following a proteinase K pretreatment *α*-syn aggregations were visible in all analyzed double-mutant animals and in two out of four analyzed monomutants. These findings could only be observed in 8 m- but not in 3 m-old animals. This is in agreement with previous studies of mouse models [24, 25] and humans [26] where *α*-syn aggregations only occurred in the spinal cord but not in the substantia nigra. These results hint towards an early involvement of the spinal cord in the origination of PD.

In addition, inflammatory processes were increased in the transgenic mice relative to LM. Transgenic mice exhibit higher numbers of GFAP-positive astrocytes and activated microglia. Furthermore, the lysosomal cystein proteases CATS and CATX are more abundantly expressed in the cervical spinal cord of BAsyn and BAsyn/PaKO compared to LM. These inflammatory processes are accompanied by ongoing cell death, as shown by FluoroJade and Gallyas staining. Analyzes of adjacent slices could identify most cells positive for cell death staining as astrocytes. 

Interestingly, these changes only occur during aging, as 3 m-old mice are not affected. This reinforces the hypothesis that early PD pathology builds up during aging [27]. All four BAsyn/PaKO mice analyzed displayed the pathological impairments described above whereas the appearance of inflammation and cell death occurred only in two out of four BAsyn animals. This suggests that the effect of the overexpression of *α*-syn is enhanced by the deletion of the parkin gene. This may possibly be explained by the fact that parkin is involved in the degradation of alpha-synuclein [5]. However, the combination of both was not seen to enhance cell death in astrocytes in similar model systems [28].

In addition, the double-mutants exhibit high mitochondrial damage in astrocytes and oligodendrocytes of the cervical spinal cord, indicating a severe glial reaction that is associated with the appearance of astrocytic cell death. The highest mitochondrial damage occurs in oligodendrocytes, even though they do not express the transgene. Inclusions of *α*-syn in oligodendrocytes from transgenic mouse models of MSA which overexpress alpha-synuclein have been shown to participate in neurodegeneration [14, 15]. Interestingly, our data implicate that the expression of the transgene in oligodendrocytes is not a necessary requirement for the presence of damaged mitochondria in the same cell type. In contrast, only minor ultrastructural disturbances were detected in motor neurons, which also do not express the transgene. The damage in neurons and, more pronounced, in oligodendrocytes might be triggered by neighbouring impaired astrocytes. Oligodendrocytes may be more prone to mitochondrial damage than neurons which are already visible in a higher number of damaged mitochondria in LM (Figure 3(g)). The transgenic *α*-syn might start a pathological cascade in astrocytes. Under pathological conditions astrocytes can become reactive glia, releasing cytokines, and reactive oxidative molecules. These cells then fail to provide normal support to neurons [10, 29], thereby causing mitochondrial damage in these cells. Oligodendrocytes do not seem to play a role in promoting inflammation although, like neurons, they are damaged by inflammatory processes. These data implicate that the *α*-syn expression mimics physiological effects which may be relevant to PD or other diseases related to alpha-synuclein expression. 

All pathological findings—gliosis, inflammation, cell death, and mitochondrial damage—occurred in the AH, ZI, LF, and in the AF of the cervical spinal cord of the transgenic mice. This may resemble mouse models for *α*-synucleinopathies, where astrogliosis emerged in the spinal cord [30, 31]. No changes could be observed in the posterior funiculus (PF) or the posterior horn (PH). The detected alterations are in accordance with the appearance of *α*-syn aggregations. Consisting mainly of motor neurons, AH, and AF are the most affected regions leading to motor impairments. Though motor neurons are not structurally affected, they are surrounded by altered glial cells which may lead to changes in neuronal function and, therefore, initiate PD symptoms. This hypothesis is strengthened by the data of the footprint analyses. At the age of 8 m both transgenic lines display motor impairments like gait disturbances very similar to the symptoms in PD patients [32]. Furthermore, the Elevated Plus Test was used to measure the tendency of mice to explore a new environment. The test is based on the natural aversion of mice for open and elevated areas, as well as on their natural spontaneous exploratory behavior [33]. We found severe deficits in the performance of both mouse lines compared to the LM. The unwillingness of the mice to explore a new environment, measured by this test, could be due to their gait disturbances. 

Our mouse models express the *α*-syn under the control of the BA promoter, leading to the expression of mutated *α*-syn in astrocytes. Similarly, the parkin gene is knocked out in every cell. Therefore, this model reflects the situation in humans, as humans express parkin and *α*-syn in almost every tissue. In spite of this general expression pattern, we found the described pathology exclusively in the mouse spinal cord, resembling the situation in early PD patients.

## 5. Conclusion

We demonstrated that the spinal cord of *α*-syn transgenic mice is more severely affected than higher brain regions. The results support the hypothesis that *α*-syn overexpression induces astrocyte-derived toxicity, leading to a pathology of the spinal cord that also involves oligodendrocytes and eventually neurons. This may lead to impaired motor coordination. The nature of the genes involved and the pathological changes indicate a resemblance of the mouse models to MSA or PD. Therefore, it is tempting to speculate that pathological changes in nonneuronal cells may contribute to *α*-synucleinopathies.
